# The Proteomic Profiling of a Platelet-Enriched Fraction Identifies an Inflammatory and Immune-Related Signature Associated with Non-Response to Treatment with Plasma Rich in Growth Factors in Knee Osteoarthritis

**DOI:** 10.3390/biom16101482

**Published:** 2026-10-12

**Authors:** Alessandra Colombini, Vincenzo Raffo, Elena De Vecchi, Laura Pandolfi, Emiliano Marasco, Giulio Grieco, Simona Landoni, Margot Lo Pinto, Simone Dario Scilabra, Laura de Girolamo

**Affiliations:** 1 Orthopaedic Biotechnology Lab, IRCCS Galeazzi-Sant’Ambrogio Hospital, 20157 Milan, Italy; giuliogrieco98@gmail.com (G.G.); simona.landoni@grupposandonato.it (S.L.); laura.degirolamo@grupposandonato.it (L.d.G.); 2 Department of Precision and Regenerative Medicine and Ionian Area (Di. Me. Pre-J), University of Bari, 70124 Bari, Italy; v.raffo@phd.uniba.it; 3 Laboratory of Clinical Chemistry and Microbiology, IRCCS Galeazzi-Sant’Ambrogio Hospital, 20157 Milan, Italy; elena.devecchi@grupposandonato.it (E.D.V.); laura.pandolfi@grupposandonato.it (L.P.); 4 Division of Rheumatology, Bambino Gesù Children’s Hospital, 00165 Rome, Italy; emiliano.marasco@opbg.net; 5 Proteomics Group of Ri. MED Foundation, Research Department IRCCS ISMETT (Istituto Mediterraneo per i Trapianti e Terapie ad Alta Specializzazione), 90133 Palermo, Italy; mlopinto@fondazionerimed.com (M.L.P.); sdscilabra@fondazionerimed.com (S.D.S.)

**Keywords:** knee osteoarthritis, plasma rich in growth factors (PRGF), proteomics, immunoglobulin A, biomarkers, inflammation, orthobiologics

## Abstract

Background: The use of plasma rich in growth factors (PRGF) is a promising regenerative treatment for knee osteoarthritis (OA), but clinical response is heterogeneous. This study aimed to identify molecular features and explore clinical factors associated with PRGF non-response and explore potential biomarkers for patient stratification. Methods: One hundred patients with knee OA (Kellgren–Lawrence II–III) treated with two intra-articular PRGF injections were evaluated at baseline and 6 months. Clinical response was defined by a ≥8-point improvement in overall knee injury and osteoarthritis outcome score (KOOS). Exploratory proteomic profiling was performed in a subgroup of 33 patients (13 responders and 20 non-responders) using platelet-rich plasma-derived platelet lysate (PRP-PL) generated from the same blood-derived preparation used for treatment. Samples were analyzed by data-independent acquisition mass spectrometry, followed by a bioinformatic analysis of differential proteins and enriched pathways. To further investigate selected proteomic findings, IgA levels were quantified in PRP-PL from the entire cohort. Results: Responders showed significant improvements in pain, overall KOOS, and functional outcomes. Proteomic analysis identified a non-responder signature characterized by increased immunoglobulin chains, complement-related proteins, acute-phase molecules, extracellular matrix remodeling proteins, and metabolic stress markers. Functional enrichment revealed the activation of immune, inflammatory, and tissue remodeling pathways. IgA levels in PRP-PL were significantly higher in non-responders, consistent with the immunoglobulin-related proteomic findings. Conclusions: PRGF non-response is associated with an inflammatory and immune-related phenotype. Higher IgA levels in PRP-PL were associated with reduced treatment responsiveness and may help identify a biological phenotype less likely to benefit from PRGF treatment.

## 1. Introduction

Knee osteoarthritis (OA) is a prevalent chronic musculoskeletal disorder characterized by cartilage degradation, synovial inflammation, subchondral bone remodeling, and persistent pain, leading to functional impairment and reduced quality of life in adults worldwide [1,2,3]. Conventional management strategies, including physical therapy, non-steroidal anti-inflammatory drugs (NSAIDs), and intra-articular corticosteroids, primarily provide symptomatic relief without directly addressing the underlying degenerative and inflammatory mechanisms driving disease progression [4].

To overcome these limitations, regenerative strategies such as the use of plasma rich in growth factors (PRGF) and other autologous platelet-derived preparations have gained increasing attention. PRGF is rich in bioactive proteins such as platelet-derived growth factor (PDGF), transforming growth factor-β (TGF-β), and vascular endothelial growth factor (VEGF), which can modulate inflammation, promote tissue repair, and stimulate anabolic processes in the osteoarthritic joint [5,6]. Randomized clinical trials have demonstrated that intra-articular PRGF injections improve pain and functional outcomes compared with placebo or hyaluronic acid, showing a favorable safety profile [7,8].

Despite these encouraging results, treatment response to PRGF is heterogeneous, with a subset of patients experiencing limited or no clinical improvement. Recent clinical evidence continues to refine our understanding of these therapies. A prospective double-blind multicenter randomized controlled trial demonstrated that combining intraosseous and intra-articular PRGF infiltrations produced superior improvements in pain, function, and quality of life at 12-month follow-up compared with intraosseous saline plus intra-articular PRGF, suggesting that the direct targeting of subchondral bone may enhance outcomes in severe knee OA patients [9].

Beyond clinical outcomes, recent studies have explored biomarker associations with PRGF efficacy. Intra-articular PRGF was shown to reduce the serum levels of Coll2-1, a biomarker of cartilage degradation, correlating with clinical improvements, suggesting that biological profiles may underlie inter-individual differences in response [10,11]. These findings underscore the need to further investigate the molecular features associated with non-responsiveness, which may help elucidate the biological mechanisms underlying differential clinical responses and provide a basis for future validation studies.

To unravel this biological complexity, proteomic profiling offers a powerful approach to characterize the network of inflammatory, metabolic and immune mediators involved in knee OA [12,13].

Therefore, this study primarily aimed to characterize proteomic differences between responders and non-responders to PRGF therapy using the high-throughput profiling of cytokines, chemokines, and acute-phase proteins in platelet-rich plasma-derived platelet lysate (PRP-PL), while clinical and demographic variables were explored as potential predictors of treatment response. Integrating biological and clinical information may contribute to biomarker discovery, optimize treatment selection, and support more personalized treatment strategies in knee OA.

## 2. Materials and Methods

### 2.1. Enrollment of Patients

This study was conducted in accordance with the Declaration of Helsinki and approved by San Raffaele Hospital Ethics Committee (registered under number 6/int/2018 for regenerative medicine procedure waste material, approval date: 8 February 2018). All the participants provided signed informed consent.

Patients who came to the attention of the RE.GA.IN.^®^ Center of the IRCCS Galeazzi Sant’Ambrogio Hospital, aged over 18 years, with a diagnosis of early knee osteoarthritis (KL II and III) were enrolled. A clinician collected the patients’ medical history and performed a clinical evaluation before treatment. The clinical, risk-factor-related and algo-functional data were collected before the treatment and then at 6 months of follow-up by the same clinician.

### 2.2. PRGF Preparation and Injection

Each patient underwent two injections of CaCl_2_-activated PRGF (plasma rich in growth factors), prepared from PRP, obtained using the Endoret^®^ system (BTI, Vitoria, Alava, Spain) and administered at an interval of two weeks. To ensure a highly standardized product composition, Fraction 2 was isolated according to the manufacturer’s commercial protocol; this closed system is explicitly designed to yield a reproducible, leukocyte-free and erythrocyte-free pure platelet-rich plasma matrix [14,15]. Baseline platelet count was assessed before the first injection with a Sysmex XN-2000 automated hematology analyzer (Sysmex, Kobe, Japan).

### 2.3. Assessment of Clinical Responsiveness

Algo-functional outcomes, such as the Visual Analog Scale (VAS; a psychometric measure of subjective pain intensity on a continuous line) [16], EuroQol Visual Analogue Scale (EQ VAS) [17] and the Knee injury and Osteoarthritis Outcome Score (KOOS; a patient-reported outcome measure of knee symptoms and function encompassing other symptoms, pain, activities of daily living, sport, and knee-related quality of life) [18], were used as reliable tests to assess the clinical outcome of the procedure.

The overall KOOS was calculated as the mean of the 42 KOOS items and linearly transformed to a 0–100 scale, with higher scores indicating better health status.

Treatment response was defined a priori as a ≥8-point increase in the overall KOOS from baseline to 6 months, based on the responder threshold reported by Faber et al. [19].

### 2.4. Proteomic Analysis

The platelet-enriched samples used for proteomic analysis were generated from aliquots of the same PRP preparation used for the clinical treatment. PRP samples were subjected to five consecutive freeze–thaw cycles, following the protocol described by Strandberg et al. [20]. Briefly, samples were frozen at −80 °C and thawed at room temperature during each cycle to induce platelet disruption and the consequent release of platelet-derived proteins and growth factors (PRP-PL). The same freeze–thaw procedure was applied to all samples to minimize pre-analytical variability. PRP-PL was used as a standardized analytical matrix to investigate the patient-specific biological characteristics of the platelet preparation, thereby allowing for inter-individual molecular profiling under controlled experimental conditions.

The PreOmics iST-BCT kit (PreOmics GmbH, Martinsried, Germany) was used to process 20 µg of protein per sample, according to the manufacturer’s instructions. Briefly, 50 µL of LYSE-BCT buffer was added to each sample before heating at 95 °C for 10 min with agitation at 1200 rpm. After cooling to room temperature, trypsin digestion buffer was added, and samples were incubated at 37 °C for 3 h under continuous shaking at 1200 rpm. The digestion was terminated by the addition of the provided stop buffer, and the resulting supernatant was purified using the supplied filter cartridges. Peptides were eluted twice with 150 µL of elution buffer, dried using a speed vacuum concentrator, and resuspended in 150 µL of LC buffer. Peptide concentrations were measured using a NanoDrop spectrophotometer (Thermo Fisher Scientific, Waltham, MA, USA), and 350 ng per sample was injected for mass spectrometry analysis.

NanoLC separation was performed using a Vanquish Neo UHPLC system coupled online to an Orbitrap Astral mass spectrometer (both from Thermo Fisher Scientific, Waltham, MA, USA) equipped with a 75 µm × 150 mm DNV PepMap Neo column (Thermo Fisher Scientific). Peptides were separated using a 20-min binary gradient consisting of water (solvent A) and acetonitrile (solvent B), both containing 0.1% formic acid.

Data-independent acquisition (DIA) was performed using a full MS1 scan range of 380–980 *m*/*z*, followed by 199 sequential DIA windows of 3 *m*/*z* width without overlap. Full MS scans were acquired at a resolution of 240,000, with an automatic gain control (AGC) target of 5 × 10^6^ and a maximum injection time of 5 ms. DIA scans were acquired at a fixed resolution of 80,000, with an AGC target of 5 × 10^4^ and a maximum injection time of 5 ms. The scan range was set to 100–1000 *m*/*z*, with higher-energy collisional dissociation (HCD) collision energy set at 25%.

### 2.5. Bioinformatics and Statistical Analysis of Proteomic Data

Raw data were analyzed using DIA-NN software (v2.0.2) with a predicted spectral library generated from an in silico digestion of the human UniProt reference proteome (UP000005640_9606, downloaded 3 April 2025). Enzymatic cleavage was set to trypsin specificity (K/R), allowing for up to two missed cleavages and a minimum peptide length of six amino acids.

Peptide- and protein-level false discovery rates (FDRs) were controlled at 1%. Label-free protein abundances were quantified using the QuantUMS (high-precision) strategy, with RT-dependent cross-run normalization and match-between-runs (MBR) enabled. Statistical analyses were performed in Perseus (v2.0.9.0; Max Planck Institute of Biochemistry, Martinsried, Germany). Protein intensities were log2-transformed, and proteins with valid values in at least 70% of samples in at least one group were included in the analyses. Differential abundance between responders and non-responders was assessed using a two-sided *t*-test without imputing missing values. A permutation-based FDR threshold of 0.05 was evaluated; however, proteins were selected for exploratory analysis based on an unadjusted *p*-value < 0.05, with no fold-change threshold. These results were visualized in a volcano plot. For principal component analysis (PCA), missing values in the filtered protein matrix were imputed from a normal distribution using a width of 0.3 and a downshift of 1.8.

Functional enrichment and protein–protein interaction (PPI) network analyses were conducted using Metascape to identify significantly enriched biological processes and pathways. Interaction networks were constructed using the Physical Core database, including only high-confidence physical protein–protein interactions.

### 2.6. Quantification of IgA

Immunoglobulin A (IgA) concentrations were measured in PRP-PL samples using the Atellica^®^ Solution clinical chemistry and immunoassay analyzer (Siemens Healthineers, Erlangen, Germany). Analysis was performed according to the manufacturer’s instructions, using the corresponding validated reagent kits and standardized calibration procedures validated for plasma samples. Internal quality control materials were analyzed routinely to ensure the accuracy and precision of the measurements throughout this study.

### 2.7. Statistical Analysis of Clinical and Outcome Data

Statistical analysis was conducted for exploratory purposes to identify potential clinical and demographic factors associated with treatment response, defined as a dichotomous variable (responder vs. non-responder).

Continuous variables were assessed for distributional assumptions using the Shapiro–Wilk test, supported by the visual inspection of histograms and Q-Q plots (Figures S1 and S2). For normally distributed continuous variables, comparisons between responders and non-responders were performed using an unpaired two-tailed Student’s *t*-test, whereas non-normally distributed variables were analyzed using the Mann–Whitney U test. The continuous variables evaluated included age, body mass index (BMI), and pre-infiltration platelet count.

Categorical variables, including sex, time of diagnosis, trauma history, smoking habit, alcohol consumption, drug use, previous treatments, previous surgery, supplement use, and concomitant pathologies (metabolic, autoimmune, diabetes, cardiovascular), were compared between groups using Pearson’s chi-square test. Fisher’s exact test was applied when expected cell frequencies were insufficient to meet chi-square assumptions. Smoking habit and concomitant pathologies were included as clinically relevant baseline factors because smoking has been investigated in relation to knee OA, while comorbidity burden has been associated with pain and functional outcomes in patients with knee and/or hip OA [21,22].

Variables showing a potential association with treatment response in the univariate analysis (exploratory threshold *p* ≤ 0.20), together with clinically relevant covariates, were considered candidates for multivariable analysis. This permissive screening approach was used for exploratory variable identification rather than as an automatic variable-selection procedure.

A multivariable binary logistic regression model was subsequently fitted to estimate the independent association between selected variables and treatment response. Given the limited number of outcome events, the model was intentionally restricted to a parsimonious number of covariates to minimize the risk of overfitting. The results from logistic regression were reported as odds ratios (ORs), with corresponding 95% confidence intervals (95% CIs) and *p*-values. An additional model including age adjustment was performed to assess the potential influence of age as a confounding factor. The analytical cohort comprised 100 patients, including 54 responders and 46 non-responders. Responders were coded as the outcome event; therefore, the first logistic regression model included 54 outcome events and two covariates, corresponding to 27 events per covariate, while the age-adjusted model included 54 outcome events and three covariates, corresponding to 18 events per covariate.

For the outcome measures analysis, the distribution of baseline and 6-month KOOS values was assessed using the Shapiro–Wilk test, supported by the visual inspection of the data distributions. Baseline and 6-month KOOS values were compared between responders and non-responders using an unpaired two-tailed Student’s *t*-test or the Mann–Whitney U test, according to data distribution. Changes in KOOS from baseline to 6 months were also compared between groups using the Mann–Whitney U test.

To further assess 6-month KOOS while accounting for potential differences in baseline KOOS, a continuous-outcome sensitivity analysis was performed using an ANCOVA-type linear regression model. Six-month KOOS was used as the dependent variable, with baseline KOOS included as an adjustment variable. Smoking and concomitant pathologies were included as clinical covariates, and a further model additionally adjusted for age. Regression coefficients (β), 95% confidence intervals (95% CIs), and *p*-values were reported. This analysis was performed to complement the dichotomous responder analysis and to reduce the potential influence of baseline severity and outcome dichotomization.

All statistical tests were two-sided, and a *p*-value ≤ 0.05 was considered statistically significant for the final multivariable models and continuous-outcome analyses. Statistical analyses were performed using R software (version 2026.01.1+403, R Core Team, Vienna, Austria).

## 3. Results

### 3.1. Description of Enrolled Patients

A total of 100 patients were enrolled. Of these, 54 patients were responders (defined by a ≥8-point improvement in overall KOOS) and 46 non-responders to the PRGF treatment. Table 1 shows the features of the enrolled patients.

Age showed a distribution compatible with normality in both groups and was therefore compared using Student’s *t*-test, whereas BMI and pre-infiltration platelet count showed non-normal distributions in at least one of the groups and were consequently compared using the Mann–Whitney U test.

In the univariate analysis, none of the continuous variables showed statistically significant differences between responders and non-responders: age (unpaired two-tailed Student’s *t*-test, *p* = 0.095), BMI (Mann–Whitney U test, *p* = 0.572), and pre-infiltration platelet count (Mann–Whitney U test, *p* = 0.233).

Categorical variables, including sex, diagnostic category, trauma history, smoking habit, alcohol consumption, medication use, previous treatments, previous surgery, supplement use, and presence of concomitant pathologies, were compared using Pearson’s chi-square test, applying Fisher’s exact test when expected frequencies did not meet the assumptions of the chi-square test. None of the categorical variables reached conventional statistical significance. In particular, females represented 42.6% of responders and 30.0% of non-responders, whereas males represented 57.4% and 70.0%, respectively. Thus, the distribution of sex did not indicate a higher proportion of female patients among non-responders. A history of trauma was reported in 16.7% of responders and 13.1% of non-responders, with no statistically significant difference between groups.

However, the presence of concomitant pathologies showed the strongest exploratory signal (χ^2^ test, *p* = 0.097) and was therefore included in the multivariable model together with the variable smoking, which was retained as clinically relevant.

In the first multivariable logistic regression model, including smoking and concomitant pathologies, smoking habit was not significantly associated with treatment response (OR = 1.60, 95% CI 0.65–4.10; *p* = 0.315). Concomitant pathologies showed an exploratory association with treatment response (OR = 2.34, 95% CI 1.03–5.49; *p* = 0.045). However, after adjustment for age, this association was attenuated and no longer statistically significant (OR = 1.95, 95% CI 0.81–4.84; *p* = 0.140). Age was also not significantly associated with response (OR = 1.02 per yearly increase, 95% CI 0.98–1.06; *p* = 0.268). Smoking remained non-significant after age adjustment (OR = 1.60, 95% CI 0.65–4.14; *p* = 0.315).

Overall, the clinical and demographic analysis did not identify robust independent factors associated with treatment response. The exploratory association observed for concomitant pathologies in the initial model was not maintained after age adjustment.

### 3.2. Clinical Outcomes and Treatment Response at 6 Months

**Figure 1:**
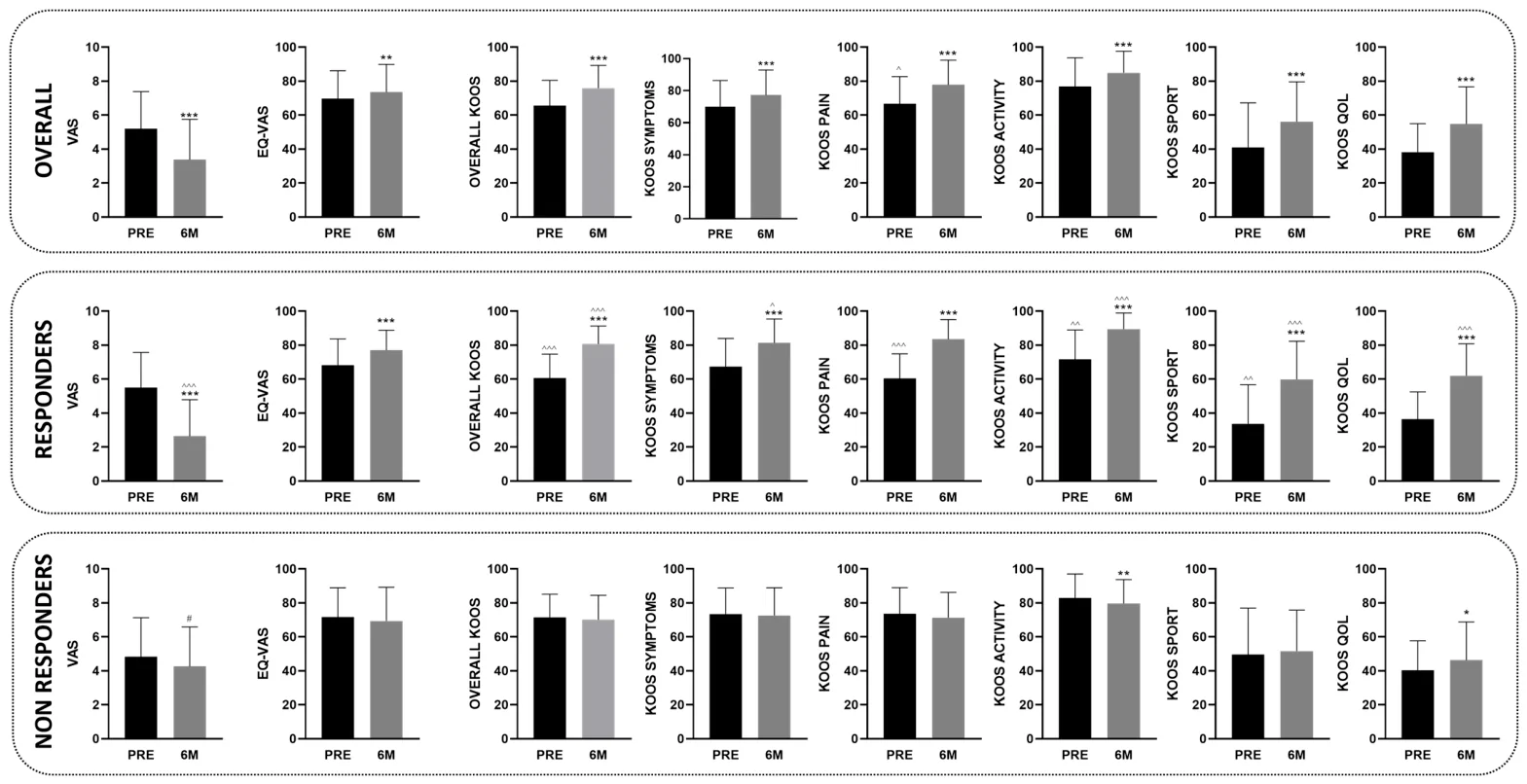
Clinical outcomes after PRGF treatment in the overall cohort (N = 100 patients) and responder/non-responder subgroups. Changes in patient-reported outcomes from baseline (PRE) to 6 months after PRGF treatment (6M) in the overall population (**upper panel**), responders (**middle panel**), and non-responders (**lower panel**). Clinical outcomes included the Visual Analog Scale (VAS) for pain, EuroQol Visual Analogue Scale (EQ-VAS) and overall Knee injury and Osteoarthritis Outcome Score (KOOS) and subscales (symptoms, pain, activities of daily living, sport/recreation, and knee-related quality of life). Data are presented as the mean ± standard deviation. * Statistically significant changes compared with baseline (PRE); ^ statistically significant differences compared with non-responders (NRs). # Indicates a statistical trend (*p* < 0.10). Statistical significance was defined as * *p* ≤ 0.05. ** *p* ≤ 0.01, *** *p* ≤ 0.001; ^ *p* ≤ 0.05. ^^ *p* ≤ 0.01, ^^^ *p* ≤ 0.001.

Overall, a general improvement in clinical outcomes was observed from baseline to 6 months across the entire cohort of 100 patients. Pain, as measured by the VAS, showed a reduction over time, accompanied by improvements in EQ-VAS, overall KOOS and in all KOOS subscales (pain, symptoms, activities of daily living, sport/recreation, and quality of life) (Figure 1). At 6 months, VAS scores showed a significant negative correlation with the KOOS pain subscale (Spearman’s r = −0.59, 95% CI = −0.71–−0.44; *p* < 0.0001).

The responder subgroup showed greater improvements across several clinical outcome measures at 6 months compared with the non-responder subgroup. The between-group differences were the most evident for overall KOOS, symptoms, activities of daily living, sport/recreation and quality of life, as well as for VAS pain scores. Baseline overall KOOSs, as well as the pain, activities of daily living and sport/recreation subscale scores, were lower in responders than in non-responders, indicating greater baseline impairment in the responder group. Responders subsequently showed greater improvement over time, whereas changes were less pronounced in non-responders.

Because baseline KOOS differed between the two groups, the relationship between baseline and 6-month KOOS was further examined using a continuous-outcome sensitivity analysis adjusted for baseline KOOS, as described below.

Baseline and 6-month KOOS values were further examined to assess the potential influence of baseline severity on the responder classification. Responders had significantly lower baseline overall KOOS values than non-responders (mean ± SD: 60.63 ± 14.11 vs. 71.36 ± 13.76; median [IQR]: 62.50 [49.70–71.88] vs. 72.62 [65.03–80.21]; Mann–Whitney U test, *p* = 0.00026). At 6 months, overall KOOS was higher in responders than in non-responders (80.70 ± 10.60 vs. 70.16 ± 14.28). The mean change in KOOS from baseline to 6 months was +20.07 ± 9.37 in responders and −1.20 ± 6.03 in non-responders (Table 2).

To complement the dichotomous responder analysis, a continuous-outcome sensitivity analysis was performed using an ANCOVA-type linear regression model with 6-month KOOS as the dependent variable and baseline KOOS as an adjustment variable. In the model including baseline KOOS, smoking, and concomitant pathologies, baseline KOOS was independently associated with 6-month KOOS (β = 0.521, 95% CI 0.366–0.675; *p* < 0.001), whereas smoking (β = 2.13, 95% CI −2.94–7.21; *p* = 0.406) and concomitant pathologies (β = 1.02, 95% CI −3.65–5.69; *p* = 0.666) were not significantly associated with 6-month KOOS. Further adjustment for age produced similar findings: baseline KOOS remained significantly associated with 6-month KOOS (β = 0.526, *p* < 0.001), whereas age (*p* = 0.548), smoking (*p* = 0.404), and concomitant pathologies (*p* = 0.848) were not significantly associated with the outcome. These findings provide complementary evidence to the responder-based analysis and indicate that the exploratory association between concomitant pathologies and treatment response observed in the initial logistic regression was not supported when KOOS was analyzed as a continuous outcome while accounting for baseline KOOS.

### 3.3. Proteomic Profiling of PRP-PL

Proteomic profiling was initially conducted in a discovery cohort of 33 patients, selected based on the availability of suitable biological samples collected during the initial phase of this study. This cohort included 21 males and 12 females, of whom 13 were responders and 20 were non-responders to PRGF treatment. The proteomic findings identified in the discovery cohort were subsequently assessed in the remaining 67 patients, constituting the independent biomarker assessment cohort, for a total study population of 100 patients.

**Figure 2:**
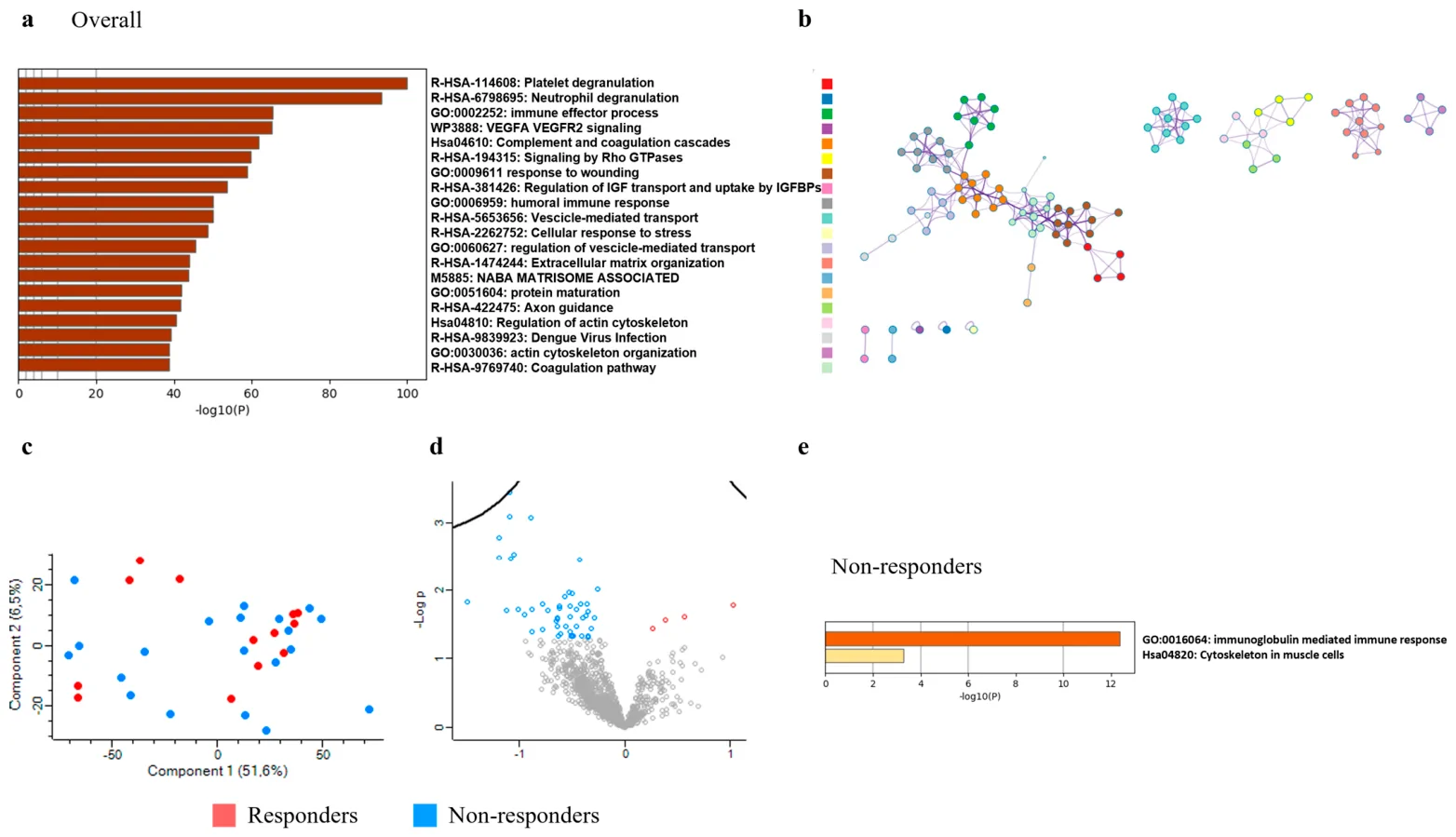
Proteomic profiling and functional analysis of PRP-PL samples. Proteomic profiling was conducted on exploratory cohort of patients. (**a**) Pathway enrichment analysis of proteins expressed in overall cohort, ranked by –log10(P). (**b**) Network visualization of enriched biological processes. (**c**) Principal component analysis (PCA) showing no clear separation between responders (red) and non-responders (blue). (**d**) Volcano plot of differential protein abundance showing proteins with nominally higher abundance in responders (red) and non-responders (blue) at unadjusted *p*-value < 0.05. Grey dots indicate proteins with an unadjusted *p*-value ≥ 0.05. The *x*-axis shows log2(responder/non-responder) abundance ratio. The black hyperbolic curves indicate the permutation-based FDR threshold of 0.05; no proteins met this threshold. (**e**) Pathway enrichment analysis in non-responders.

A total of 2821 proteins were identified in at least one sample of the PRP-PL of the discovery cohort of patients. After filtering for valid values in at least 70% of samples in at least one group, 1445 proteins were retained for downstream analysis. The multiple panels in Figure 2 summarize the proteomic and functional analysis of PRP-PL samples.

Panel a shows the enrichment in key pathways related to the proteins expressed in the overall cohort of patients, ranked by –log10(P). The top pathways include platelet-related pathways, neutrophil degranulation, immune response, VEGFA/VEGFR2 signaling, complement activation, vesicle-mediated transport and matrix remodeling. Panel b presents a network visualization of enriched biological processes, illustrating the functional clustering of proteins. Nodes are colored by pathway category, highlighting a dense central network where immune and vesicle-mediated pathways are physically interconnected, contrasted by isolated, tightly clustered modules representing matrix and cytoskeletal structural processes. Panel c includes a principal component analysis (PCA) plot, where non-responder samples (blue) do not cluster distinctly from responders (red).

PC1 and PC2 explained 51.6% and 6.5% of the total variance, respectively. Panel d shows a volcano plot of differential protein abundance, highlighting 4 proteins with nominally higher abundance in responders (red) and 48 in non-responders (blue) at an unadjusted *p*-value < 0.05; none met the permutation-based FDR threshold of 0.05.

Panel e provides a secondary pathway enrichment analysis in non-responders, emphasizing immunoglobulin-mediated signaling.

Table 3 shows the proteins upregulated in non-responders in comparison with responders.

In the non-responder group, PRP-PL revealed a predominance of immunoglobulin light and heavy chains (IGLV, IGKV, IGHV, IGLC), suggesting increased humoral immune activity. This finding may reflect an immune-mediated phenotype associated with non-responsiveness, consistent with an inflammatory OA endotype associated with increased pain and accelerated structural progression [23,24]. Whether this profile reflects or contributes to the inflammatory joint microenvironment remains to be established.

Additional findings included elevated levels of inflammatory and pro-catabolic proteins, such as SAA4 and C1QC, reflecting chronic inflammation, complement activation, and macrophage-driven tissue catabolism. Furthermore, the elevated levels of SCGB3A1, a cytokine-like protein involved in immune regulation, further underscore the complex, dysregulated inflammatory cascade that characterizes the patients refractory to PRGF treatment.

Beyond the immune-related proteins, non-responders exhibited significant alterations in PRP-PL in proteins associated with joint structural architecture, including the upregulation of ECM-associated proteins such as TNXB, FBN1, and ITGA2. These changes were accompanied by alterations in proteins associated with metabolic, mitochondrial, and redox-related pathways. In particular, the increased abundance of PGAM1, PYGL, and IMMT was observed, together with changes in proteins involved in redox homeostasis, including GSS and BLVRB. Overall, these findings identify a distinct metabolic-, mitochondrial-, and redox-related proteomic signature in non-responders. Importantly, these protein-level alterations represent pathway-associated changes in PRP-PL and do not by themselves demonstrate mitochondrial dysfunction, impaired energy production, oxidative stress, or altered chondrocyte metabolism within the joint.

Finally, differences were also observed in proteins involved in cell signaling, survival, and stress adaptation, including STK4, LMNA, and TMBIM1.

Taken together, these findings suggest that the non-responder phenotype is associated with a distinct proteomic signature of PRP-PL involving immune-related, extracellular matrix, metabolic and severe cellular stress-related pathways.

### 3.4. An Analysis of IgA in the PRP-PL of the Overall Cohort of 100 Patients

**Figure 3:**
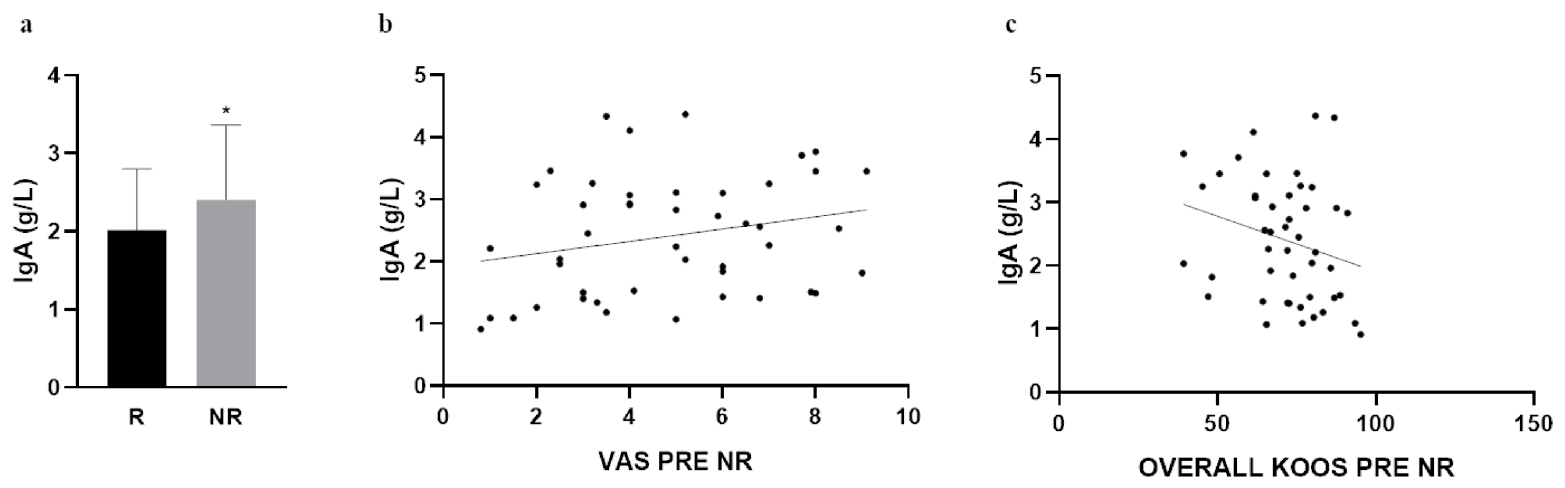
(**a**) Comparison of IgA concentrations in platelet-rich plasma-derived platelet lysate (PRP-PL) between responders (Rs) and non-responders (NRs). Bars represent mean ± standard deviation. (**b**) Correlation between IgA concentrations in PRP-PL and pre-treatment Visual Analog Scale (VAS) scores in non-responders. (**c**) Correlation between IgA concentrations in PRP-PL and pre-treatment overall Knee Injury and Osteoarthritis Outcome Score (KOOS) in non-responders. Statistical significance was set at * *p* < 0.05. *n* = 99 patients; one value for IgA was undetected in R group.

IgA concentrations were significantly higher in non-responders than in responders (2.4 ± 0.9 g/L vs. 2.0 ± 0.8 g/L, *p* = 0.0287) (Figure 3a), indicating an association between PRP-PL IgA levels and treatment response status. Within the non-responder group, IgA levels showed a non-significant positive trend toward an association with pre-treatment pain, as assessed by the VAS (r = 0.2643, *p* = 0.0759) (Figure 3b). Likewise, a non-significant negative trend was observed between IgA concentrations and pre-treatment overall KOOS in non-responders (r = −0.2823, *p* = 0.0574) (Figure 3c). Similar non-significant negative trends were also observed between IgA concentrations and overall KOOS at 6 months in the overall cohort (r = −0.1705, *p* = 0.0916) and in non-responders (r = −0.2485, *p* = 0.0959).

## 4. Discussion

The present study combined clinical and demographic assessment, untargeted proteomics and conventional laboratory biomarkers to investigate the biological characteristics associated with clinical response to PRGF treatment in patients with knee osteoarthritis. Although PRGF has demonstrated clinical benefits in mild-to-moderate knee OA, treatment response varies considerably among patients [25,26]. Moreover, reliable biomarkers associated with differential clinical response to PRGF are still lacking.

Our findings indicate that non-responders exhibit a different inflammatory and immune-related molecular profile, suggesting that host-related biological may contribute to differences in clinical response to platelet-derived therapies.

With regard to clinical and demographic characteristics, no baseline factor showed a robust independent association with treatment response. Although concomitant pathologies showed an exploratory association with response in the unadjusted analysis, this association was attenuated and no longer statistically significant after age adjustment. Moreover, the continuous-outcome sensitivity analysis did not support an association between concomitant pathologies and 6-month KOOS after adjustment for baseline KOOS, with similar findings after further adjustment for age. Overall, these findings indicate that the clinical and demographic variables evaluated in this cohort were insufficient to explain the heterogeneity of response to PRGF.

Importantly, responders had lower baseline overall KOOS values than non-responders, indicating greater baseline impairment in the responder group. This finding is relevant when interpreting the dichotomous responder definition based on a ≥8-point improvement in KOOS, because change-score definitions may be influenced by baseline severity and regression toward the mean. The additional continuous-outcome analysis provides complementary evidence by modeling 6-month KOOS while accounting for baseline KOOS. In this analysis, baseline KOOS was strongly associated with 6-month KOOS, whereas smoking and concomitant pathologies were not. These findings support a cautious interpretation of the responder-based clinical analysis and indicate that the observed exploratory association with concomitant pathologies should not be considered evidence of a definitive clinical predictor.

The exploratory nature of the clinical analysis is further relevant in view of the sample size and the variable-selection strategy. Although the multivariable models were deliberately kept parsimonious, the clinical analysis was based on 100 patients and should primarily be considered hypothesis-generating. The apparent association between concomitant pathologies and response in the initial model was not robust to age adjustment or to an analysis of KOOS as a continuous outcome. Therefore, this finding should not be interpreted as evidence of an independent effect of concomitant pathologies on PRGF responsiveness. Larger prospective cohorts will be required to determine whether any clinical or demographic characteristics are reproducibly associated with treatment response.

Age and previous trauma also warrant consideration when interpreting the clinical findings. Age was evaluated as a potential confounding factor and included in the age-adjusted logistic regression model, in which it was not significantly associated with treatment response. Similarly, the proportion of patients reporting a traumatic onset was comparable between responders and non-responders. However, because the present study did not include a younger healthy control group or younger animal controls, it cannot distinguish the effects of physiological aging from those related to osteoarthritis or previous joint injury. These factors should therefore be investigated in specifically designed comparative studies.

Overall, these findings suggest that conventional clinical and demographic variables were insufficient to explain the heterogeneous response to PRGF in this cohort, supporting the investigation of molecular features as potentially associated with treatment outcome. This is in line with previous evidence showing that clinical factors associated with response to PRP remain heterogeneous and not consistently reproducible across studies [27]. Moreover, recent ESSKA-ICRS consensus recommendations emphasize the importance of considering patient- and disease-related characteristics when selecting candidates for PRP treatment while acknowledging the limited evidence supporting robust predictive criteria [28].

The proteomic analysis revealed that non-responders to PRGF treatment were characterized by the overexpression of numerous immunoglobulin heavy- and light-chain proteins, together with molecules involved in complement activation, acute-phase response, extracellular matrix remodeling, mitochondrial metabolism and cellular stress. The functional enrichment analysis of PRP-PL proteins consistently highlighted immune-mediated pathways, suggesting that non-responders are characterized by a PRP-PL molecular profile enriched in inflammatory and immune-related proteins.

These findings are in agreement with the current concept that osteoarthritis is a heterogeneous disorder composed of different molecular endotypes [29,30], among which an inflammatory phenotype has been increasingly recognized [31]. Recent proteomic studies have demonstrated that the activation of innate and adaptive immune pathways, complement cascade, neutrophil activation and extracellular matrix degradation characterize subsets of patients with more symptomatic and progressive disease [32,33,34]. The targeted quantification of IgA in PRP-PL provides an important orthogonal confirmation of the exploratory proteomic results. Patients who failed to respond to PRGF displayed significantly higher IgA concentrations in PRP-PL than responders, supporting the enrichment in immunoglobulin-related proteins detected by mass spectrometry and extending this observation to an additional group of patients.

Because PRGF is a plasma-based preparation obtained with a standardized, leukocyte-free protocol, immunoglobulins in PRP-PL derive largely from the patient’s circulating plasma rather than from platelets or from variability in processing. This finding therefore admits two interpretations, which are not mutually exclusive. First, immunoglobulin abundance may act as a marker of the patient’s immune-related biological phenotype. Although OA has historically been considered a non-autoimmune disease, accumulating evidence indicates that adaptive immune responses contribute to disease progression. Synovial tissues from OA patients contain activated B lymphocytes and plasma cells capable of producing immunoglobulins locally [35,36]. Furthermore, immunoglobulin deposition has been demonstrated within damaged cartilage and synovial membranes [37], and extracellular matrix neoepitopes exposed by tissue injury may stimulate antibody production and sustain chronic low-grade inflammation [38,39]. In this scenario, higher immunoglobulin levels would not indicate an autoimmune process comparable to rheumatoid arthritis but rather an inflammatory OA endotype that is intrinsically less responsive to PRGF, independently of the immunoglobulins contained in the injected product [40]. Second, because immunoglobulins are delivered into the joint together with platelet-derived growth factors, they may act as an active component of the product that limits its efficacy. Immunoglobulins can promote complement activation and Fc receptor signaling [41], and IgA-containing immune complexes in particular can activate myeloid cells through FcαRI (CD89) and induce the release of inflammatory mediators [42,43], potentially counteracting the anti-inflammatory and anabolic effects of PRGF. The present observational design does not allow these possibilities to be distinguished.

Interestingly, previous investigations have also reported the presence of IgA within osteoarthritic synovial fluid and inflamed synovial tissue, supporting the concept that both systemic and local humoral immune responses participate in OA pathogenesis [35]. Although synovial immunoglobulin production is less pronounced than in inflammatory arthropathies [40], local plasma-cell infiltration and immunoglobulin deposition have been documented in osteoarthritic joints, particularly in patients presenting greater synovitis and more severe symptoms [44]. Although our study was not designed to establish a causal role for IgA, these findings raise the hypothesis that differences in immune components within autologous blood-derived products may be associated with their therapeutic efficacy and deserves further mechanistic investigation. IgA is increasingly recognized as an active regulator of mucosal and systemic immunity rather than merely a protective antibody [45,46].

Besides immunoglobulins, non-responders showed an increased abundance of proteins associated with chronic inflammation, including serum amyloid A4 and complement component C1q. The activation of the complement cascade has emerged as one of the principal mechanisms driving cartilage degeneration in OA [47]. Complement proteins promote synovial macrophage activation [48,49], chondrocyte dysfunction and extracellular matrix degradation, thereby establishing a self-perpetuating inflammatory loop [50]. Likewise, serum amyloid proteins are increasingly considered markers of chronic low-grade inflammation associated with disease severity and structural progression [51,52]. The coexistence of complement-related proteins, acute-phase proteins and immunoglobulin enrichment observed in our study is consistent with the presence of an inflammatory and immune-related molecular phenotype in non-responders. Whether this phenotype contributes to reduced response to PRGF, reflects greater disease severity, or is associated with differences in the composition of the PRP-PL preparation remains unresolved.

Our proteomic findings further demonstrated substantial alterations in proteins involved in ECM organization, metabolic and mitochondrial processes and redox regulation. The increased expression of tenascin-X, fibrillin-1 and integrin α2 indicates alterations in ECM-related proteins, while the differential expression of glycolytic enzymes, mitochondrial structural proteins, and oxidative stress regulators in non-responders highlights metabolic and redox-related changes.

Mitochondrial dysfunction and oxidative stress have recently emerged as central mechanisms contributing to chondrocyte senescence and impaired regenerative capacity [53,54,55]. Therefore, the molecular profile observed in non-responders appears to extend beyond inflammation alone and involves alterations in structural integrity, cellular metabolism and stress response.

From a clinical perspective, these findings may help characterize biological differences associated with heterogeneous responses to PRGF, despite the presence of numerous anabolic growth factors in platelet-derived products. If these signatures reflect an inflammatory or immune-related patient phenotype, they may help explain why regenerative stimuli are not associated with comparable clinical responses in all patients. However, our data do not establish whether these molecular features originate primarily from the joint environment, from patient-level biological characteristics, or from differences in PRP-PL composition. This concept is consistent with recent consensus recommendations emphasizing that the biological characteristics of both the patient and the joint should be considered in the clinical evaluation of patients considered for orthobiologic treatments [28,56,57].

The present study has several limitations. First, the proteomic investigation was performed in an exploratory subgroup with a relatively limited sample size. Although the concordant increase in IgA in PRP-PL supports the robustness of the proteomic observations, validation in larger independent cohorts is warranted. Second, IgAs were quantified in PRP-PL, whereas an analysis of synovial fluid immunoglobulins, complement activation products and cytokines could further clarify the relationship between PRP-PL composition and local inflammation. Third, comorbidities and other systemic factors may have influenced protein and immunoglobulin levels in PRP-PL and may therefore represent potential confounders of the observed associations. This consideration is particularly relevant given the signal observed for concomitant pathologies in the clinical analysis. In addition, muscle mass and periarticular muscle function were not assessed in the present study and may represent additional factors potentially influencing knee joint health and clinical response. These parameters should be considered in future studies.

Finally, the observational design precludes establishing causal relationships between immune activation and treatment failure. Specifically, this study cannot determine whether the observed PRP-PL composition reflects patient- or disease-related biological characteristics, contributes to differences in treatment response, or represents a combination of both. Moreover, because all patients received PRGF and no untreated or comparator group was included, the present study cannot determine whether the identified molecular features specifically predict benefit attributable to PRGF, as opposed to reflecting an underlying OA phenotype or natural disease trajectory.

Future studies should therefore investigate whether immunoglobulin composition within PRGF preparations differs between responders and non-responders and whether specific immune components, including IgA-containing complexes, directly modulate the biological activity of platelet-derived products. Larger external validation cohorts, together with complementary analyses of synovial fluid and/or joint tissue, will also be important to determine whether the proteomic signatures identified in this study reflect local joint processes and to further assess their clinical relevance.

## 5. Conclusions

Our study provides preliminary evidence that non-response to PRGF therapy is associated with an immune–inflammatory proteomic profile characterized by an enrichment in immunoglobulin-related proteins, complement-related pathways and inflammatory processes. The higher IgA levels observed in non-responders further support the proteomic findings and extend the observation to an additional group of patients.

The clinical and demographic analysis did not identify robust independent factors associated with treatment response. Although concomitant pathologies showed an exploratory association with response in the initial logistic regression model, this finding was not maintained after age adjustment or when KOOS was analyzed as a continuous outcome while accounting for baseline KOOS. The lower baseline KOOS observed in responders further supports a cautious interpretation of the dichotomous responder definition.

Overall, these findings identify a biological phenotype associated with clinical response but do not establish whether the observed molecular features contribute to treatment failure or reflect differences in patient- or disease-related biology and PRP-PL composition. If confirmed in larger prospective and independent cohorts, IgA and selected proteomic markers may have potential utility as biomarkers associated with clinical response and may help characterize biological heterogeneity in response among patients receiving PRGF for knee OA. Their value as predictive biomarkers of treatment benefit would require prospective evaluation in studies including an appropriate comparator group.

## Figures and Tables

**Table 1:** Features of enrolled patients.

		Overall *n* = 100	Responders *n* = 54	Non-Responders *n* = 46
Demographic, clinical and laboratory features				
Age (years; mean ± SD)		55.7 ± 11.8	57.6 ± 9.9	53.5 ± 13.9
Sex (%)	Males	63.0	57.4	70.0
	Females	37.0	42.6	30.0
BMI (Kg/m^2^; mean ± SD)		25.6 ± 4.1 ^^^	26.1 ± 4.2 ^^^	25.1 ± 3.9
Comorbidities (%)	Yes	47.0	55.6	37.0
	No	53.0	44.4	63.0
Pre-infiltration platelet count (10^3^/µL; mean ± SD)		246.7 ± 47.9 ^^^	242.2 ± 49.6	252.1 ± 45.7 ^^^
Lifestyle factors				
Smoking (%)	Yes	29.0	31.5	26.1
	No	71.0	68.5	73.9
Alcohol consumption (%)	>5 glasses	8.0	7.4	8.7
	<5 glasses	60.0	63.0	56.5
	Never	32.0	29.6	34.8
Drugs (%)	Corticosteroids/painkillers/anti-inflammatory	22.0	22.2	21.8
	No/other	78.0	77.8	78.2
Supplements (%)	Yes	63.0	66.7	58.7
	No	37.0	33.3	41.3
OA-related features				
Time from diagnosis	<1 year	29.0	37.0	19.6
	>1 year	28.0	25.9	30.4
	>3 years	43.0	37.0	50
Traumatic	Yes	15.0	16.7	13.1
	No	85.0	83.3	86.9
Previous treatments	Yes	63.0	57.4	70.0
	No	37.0	42.6	30.0
Previous surgery	Yes	63.0	33.3	28.3
	No	37.0	66.7	71.7

^^^ one missing value.

**Table 2:** Baseline and 6-month KOOSs according to treatment response.

	Non-Responders (*n* = 46)	Responders (*n* = 54)
KOOS baseline (mean ± SD)	71.36 ± 13.76	60.63 ± 14.11
KOOS baseline (median [IQR])	72.62 [65.03–80.21]	62.50 [49.70–71.88]
KOOS 6 months (mean ± SD)	70.16 ± 14.28	80.70 ± 10.60
ΔKOOS (mean ± SD)	−1.20 ± 6.03	+20.07 ± 9.37

**Table 3:** Proteins upregulated in non-responders in comparison with responders.

Category	Protein	Role in Osteoarthritis
Extracellular matrix remodeling and cell–matrix interaction	TNXB, FBN1, ITGA2, ADD1, TMEM43, SERPINA11	Altered ECM organization, cartilage structural integrity, cell–matrix adhesion, mechanotransduction and regulation of matrix turnover
Immune and inflammatory response	IGKV3-7, IGLV7-46, IGLV3-16, IGKV2-28/IGKV2D-28, IGHV3-64, IGKV1-27, IGLV5-45, IGHV4-30-2, IGHV2-26, IGHV3-74, IGHV3-38, IGKV1-8, IGLC7, IGKV1-39/IGKV1D-39, IGKV1-17, IGKV3-20, IGLV1-47, IGLV1-51, IGHV3-33, IGHV3-7, IGKV4-1, IGHV3-38-3, IGLV3-21, C1QC, SAA4, SCGB3A1	Immunoglobulin-mediated adaptive immunity, complement activation, acute-phase response and modulation of the inflammatory microenvironment
Metabolic reprogramming, mitochondrial dysfunction and redox imbalance	PGAM1, PYGL, APOF, GRHPR, IMMT, SFXN3, GSS, BLVRB	Altered glucose, glycogen and lipid metabolism associated with mitochondrial dysfunction, impaired energy production and oxidative stress
Cell signaling, survival and stress adaptation	GNG5, ECE1, STK4, LMNA, TMBIM1, ITM2B, RPLP2, SLC39A10	Dysregulated signaling pathways, apoptosis/autophagy regulation, nuclear organization, stress responses and altered zinc-dependent cellular homeostasis

ECM = Extracellular Matrix; TNXB = Tenascin XB; FBN1 = Fibrillin 1; ITGA2 = Integrin Subunit Alpha 2; ADD1 = Adducin 1; TMEM43 = Transmembrane Protein 43; SERPINA11 = Serpin Family A Member 11; IGKV = Immunoglobulin Kappa Variable; IGLV = Immunoglobulin Lambda Variable; IGHV = Immunoglobulin Heavy Variable; IGLC = Immunoglobulin Lambda Constant; C1QC = Complement C1q Subcomponent Subunit C; SAA4 = Serum Amyloid A4; SCGB3A1 = Secretoglobin Family 3A Member 1; PGAM1 = Phosphoglycerate Mutase 1; PYGL = Glycogen Phosphorylase, Liver Form; APOF = Apolipoprotein F; GRHPR = Glyoxylate Reductase/Hydroxypyruvate Reductase; IMMT = Inner Membrane Mitochondrial Protein; SFXN3 = Sideroflexin 3; GSS = Glutathione Synthetase; BLVRB = Biliverdin Reductase B; GNG5 = G Protein Subunit Gamma 5; ECE1 = Endothelin Converting Enzyme 1; STK4 = Serine/Threonine Kinase 4; LMNA = Lamin A/C; TMBIM1 = Transmembrane Bax Inhibitor Motif Containing 1; ITM2B = Integral Membrane Protein 2B; RPLP2 = Ribosomal Protein Lateral Stalk Subunit P2; SLC39A10 = Solute Carrier Family 39 Member 10.

## Data Availability

The original data presented in the study are openly available at https://osf.io/f346n/overview?view_only=aff5a1d8ff8c472bbb4d9d0ea65d9aaf (accessed on 5 October 2026).

## Supplementary Materials

The following supporting information can be downloaded at https://www.mdpi.com/article/10.3390/biom16101482/s1. Figure S1. Distribution assessment of continuous clinical variables by histograms. Histograms showing distribution of continuous variables included in exploratory analysis of treatment response, including age, body mass index (BMI), and pre-infiltration platelet count. Visual inspection was performed together with Shapiro–Wilk test to evaluate normality assumptions before selecting appropriate statistical tests; Figure S2. Quantile–quantile (Q–Q) plots for evaluation of continuous variable distributions. Q–Q plots illustrating agreement between observed and theoretical normal distributions for continuous clinical variables (age, body mass index (BMI), and pre-infiltration platelet count). These graphical assessments were used, together with Shapiro–Wilk test, to evaluate distributional assumptions for subsequent statistical analyses.

## Abbreviations

The following abbreviations are used in this manuscript:

PRGF	plasma rich in growth factors
PRP-PL	platelet-rich plasma platelet lysate
OA	osteoarthritis
KL	Kellgren–Lawrence
KOOS	knee injury and osteoarthritis outcome score
NSAIDs	non-steroidal anti-inflammatory drugs
PDGF	platelet-derived growth factor
TGF-β	transforming growth factor-β
VEGF	vascular endothelial growth factor
PROMs	patient-reported outcome measures
VAS	visual analog scale (VAS)
DIA	data-independent acquisition
AGC	automatic gain control
HCD	higher-energy collisional dissociation
FDR	false discovery rate
LFQ	label-free quantification
PPI	protein–protein interaction
IgA	immunoglobulin A
BMI	body mass index
ORs	odds ratios
CIs	confidence intervals
PCA	principal component analysis
ECM	extracellular matrix
TNXB	tenascin XB
FBN1	fibrillin 1
ITGA2	integrin subunit alpha 2
ADD1	adducin 1
TMEM43	transmembrane protein 43
SERPINA11	serpin family A member 11
IGKV	immunoglobulin kappa variable
IGLV	immunoglobulin lambda variable
IGHV	immunoglobulin heavy variable
IGLC	immunoglobulin lambda constant
C1QC	complement C1q subcomponent subunit C
SAA4	serum amyloid A4
SCGB3A1	secretoglobin family 3A member 1
PGAM1	phosphoglycerate mutase 1
PYGL	glycogen phosphorylase, liver form
APOF	apolipoprotein F
GRHPR	glyoxylate reductase/hydroxypyruvate reductase
IMMT	inner membrane mitochondrial protein
SFXN3	sideroflexin 3
GSS	glutathione synthetase
BLVRB	biliverdin reductase B
GNG5	G protein subunit gamma 5
ECE1	endothelin converting enzyme 1
STK4	serine/threonine kinase 4
LMNA	lamin A/C
TMBIM1	transmembrane BAX inhibitor motif containing 1
ITM2B	integral membrane protein 2B
RPLP2	ribosomal protein lateral stalk subunit P2
SLC39A10	solute carrier family 39 member 10
