# Supplementary material for: The Proteomic Profiling of a Platelet-Enriched Fraction Identifies an Inflammatory and Immune-Related Signature Associated with Non-Response to Treatment with Plasma Rich in Growth Factors in Knee Osteoarthritis
Source: Biomolecules. 2026 Oct 12;16(10):1482. doi: 10.3390/biom16101482 (PMC13702774; doi:10.3390/biom16101482)
Supplement: Supplementary file 1 [file biomolecules-16-01482-s001.zip › Supplementary Materials.pdf]

Supplementary Materials:

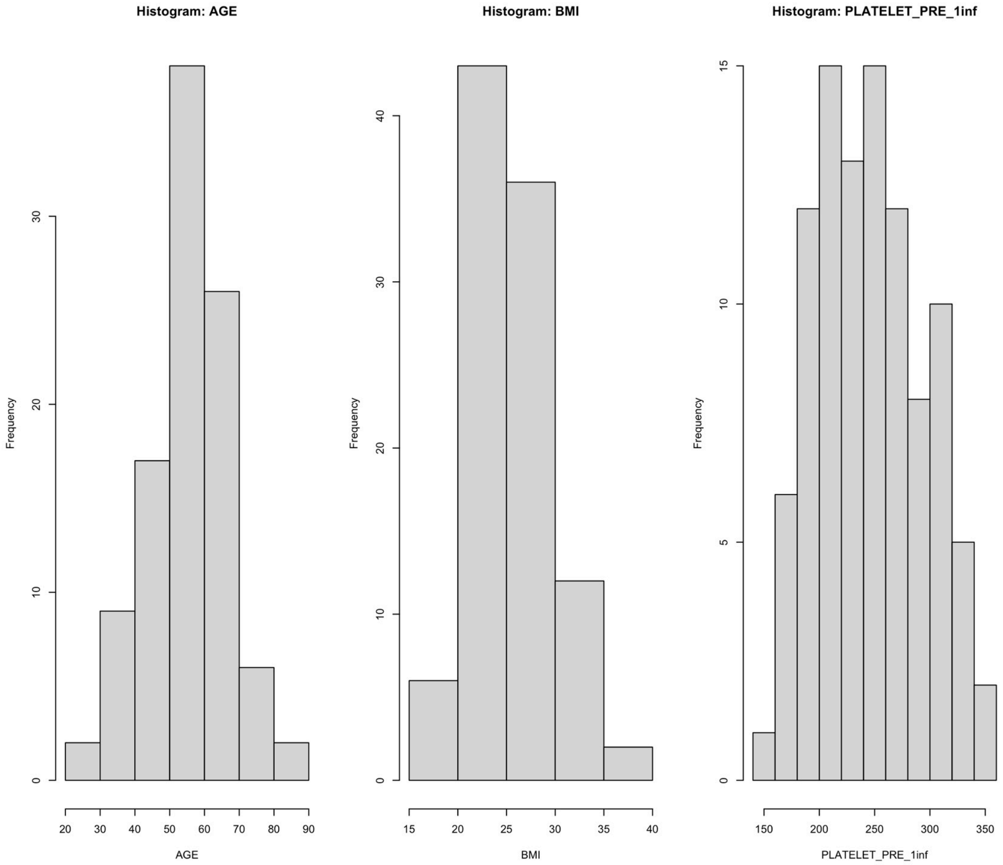

Figure S1. Distribution assessment of continuous clinical variables by histograms. Histograms showing the distribution of continuous variables included in the exploratory analysis of treatment response, including age, body mass index (BMI), and pre-infiltration platelet count. Visual inspection was performed together with the Shapiro–Wilk test to evaluate normality assumptions before selecting appropriate statistical tests.

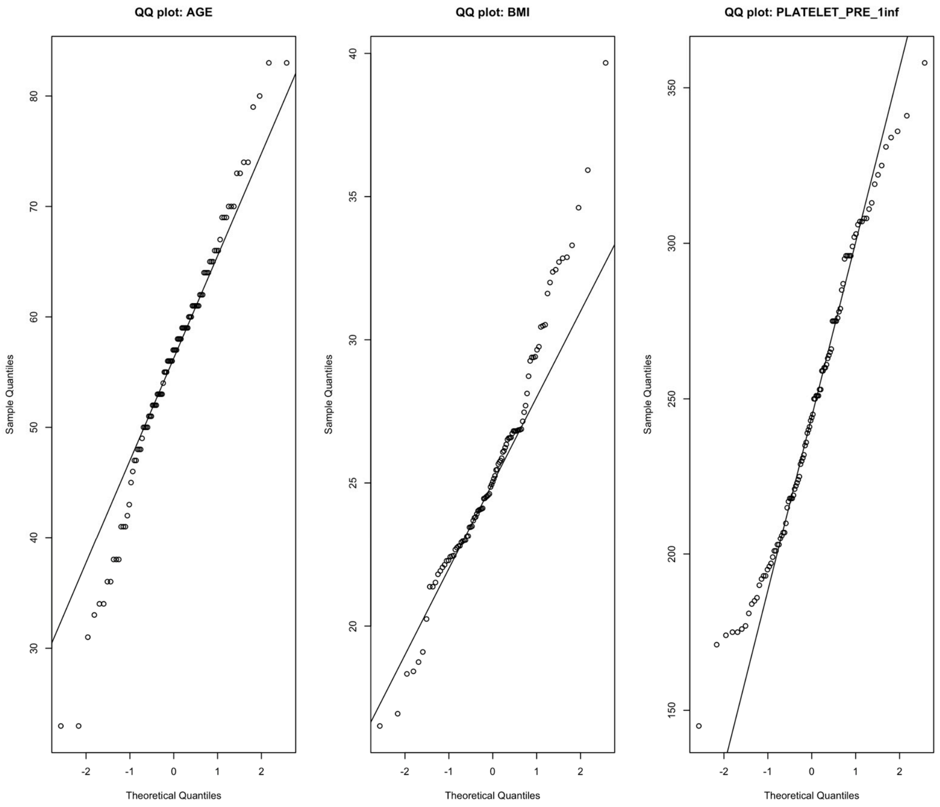

Figure S2. Quantile–quantile (Q–Q) plots for evaluation of continuous variable distributions. Q–Q plots illustrating the agreement between observed and theoretical normal distributions for continuous clinical variables (age, body mass index (BMI), and pre-infiltration platelet count). These graphical assessments were used, together with the Shapiro–Wilk test, to evaluate distributional assumptions for subsequent statistical analyses.
